# Rio Birth Cohort Study on Environmental Exposure and Childhood Development – PIPA Project

**DOI:** 10.5334/aogh.2709

**Published:** 2020-06-11

**Authors:** Carmen Ildes R. Fróes Asmus, Arnaldo Prata Barbosa, Armando Meyer, Nataly Damasceno, Ana Cristina Simões Rosa, Roberto Medronho, Antônio Jose Ledo A. da Cunha, Josino Costa Moreira, Thatiana V. R. de B. Fernandes, Marlos Martins, Ronir Raggio Luiz, Volney de Magalhães Câmara

**Affiliations:** 1 Federal University of Rio de Janeiro, School of Medicine, Rio de Janeiro, BR; 2 Federal University of Rio de Janeiro, Public Health Institute, Rio de Janeiro, BR; 3 Oswaldo Cruz Foundation, National School of Public Health, Rio de Janeiro, BR

## Abstract

**Background/Objective:**

As a developing country, Brazil presents a wide range of environmental risks that can constitute hazards to child health. The country also presents different socio-economic-cultural conditions that could be responsible for determining different vulnerability and susceptibility levels for the population, which can potentiate the effects of the environmental pollutants. The Rio Birth Cohort Study (PIPA project) is a prospective maternal-infant health study, hosted in the city of Rio de Janeiro (Southeastern Brazil), designed to investigate separate and combined effects of environmental chemical pollutants, as well as the interactions between these exposures and sociocultural environment and epigenetic patterns. This paper presents the learned lessons and strategies to address the shortcomings detected from this pilot study.

**Methods:**

The study population will be all children born at the Federal University of Rio de Janeiro Maternity Hospital from July 1st, 2020 to June 30th, 2021. The estimated population is of 2,500 children. The study will collect social, demographic, and health information from pregnant women and their children up to four years of age. Biological samples from both mothers and newborns will be collected to assess metal, pesticide and plasticizer exposure. All newborns will have their landmarks of physical, neurological, psychological, and cognitive development recorded at specific ages.

**Findings:**

A pilot study was carried out between September 2017 and August 2018, totaling 142 enrolled pregnant women, leading to 135 (95%) births and the collection of umbilical cord (126–93%,) and mother (139–98%) blood samples, as well as both mother (142–100%) and newborn (54–40%) urine samples and newborn meconium samples (117–86.7%).

**Conclusions:**

The study proposes a comprehensive assessment of pre- and postnatal exposure to environmental chemicals at multiple time points in a population living in a highly urbanized developing country. As far as we know, this is the only birth cohort in Brazil specifically designed for this purpose.

## Introduction

The Pan-American Health Organization (PAHO) estimates that 100.000 children less than five years old die each year due to environmental risks [1]. There is growing evidence that exposure to specific environmental factors or conditions during the fetal and perinatal periods can be associated with the occurrence of health disorders, not only during childhood but also in adult life [2].

Exposure to metals and pesticides during the fetal period and early years of childhood is particularly harmful to children’s health. This may lead not only to immediate effects, but also permanent and subclinical effects on brain function and structure, with losses in child development potential and late neurological and neurobehavior alterations [3,4,5,6,7]. Some pesticides present immunologic, neurologic, and mutagenic toxic action and may also act as potential endocrine disruptor, associated to a higher risk of childhood cancer [8,9,10].

As a developing country, Brazil presents a wide range of environmental risks that can constitute hazards to child health. According to Froes Asmus et al. (2016), a total of 109 studies concerning child exposure to environmental pollutants, from the fetus stage up to 11 years old, were carried out in Brazil from 1995 to 2015. Exposure to metals were reported in 72 studies, to air pollutants in 43 studies, to pesticides in 19 studies and to “others” (electromagnetic fields, coal dust, fluoride intake, refinery and chemical fertilizers and organic solvents) in six studies. Health harmful effects were reported in 74 studies. The most frequently cited effect was hospital admission for respiratory causes among children living in in areas with high concentration of air pollutants. A broad spectrum of other health effects possibly linked to pollutants were also found, such as prematurity, low Apgar scores, low birth weight, neonatal deaths, changes in cognitive function and neurobehavioral performance, congenital abnormalities and higher risk for leukemia in children less than two years old [11].

Urban population growth in Brazil determines the correlated exposure increase to urban environment pollutants. These include indoor and outdoor pollutants, such as particulates, gases and contaminants originating from production processes (buildings, commerce, industries), vehicle traffic, domestic use of industrial products (plasticizers in utensils and furniture, and pesticides to combat mosquitoes and other insects), as well as ingested food and water contaminants (metals, pesticides) [12,13].

Rio de Janeiro is the sixth most populous city in the American continent. The city is well-known by the use of large amounts of pyrethrin-derived compounds to control vector-borne arboviral diseases, such as Dengue, Zika, and Chikungunya in urban areas. Additionally, it is characterized by high violence levels, with a violent death rate of 34.9 (per 100,000.00 habitants) in 2016 [14]. Violence, in general, is part of a socioeconomic adversity environment which acts as a “toxic stress” [15]. Many studies have pointed out that childhood exposure to chronic stress may negatively impact interlinked actions of both the neurological and immunological systems [16,17,18]. Many environmental pollutants also have toxic effects on these systems. It is possible to hypothesize that violence and environmental pollutant exposure, acting synergistically since the prenatal period, may affect child neurological and immune development.

According to Landrigan and Baker (2015), birth cohorts with a medium and long period follow-up are the best epidemiologic study design to investigate possible associations between early environmental pollutant exposures (fetal or perinatal periods) and health effects in childhood and adult life [19]. Therefore, the Rio Birth Cohort study (PIPA project) aims to investigate individual and combined effects of environmental chemical pollutants, as well as the interactions between these exposures and sociocultural environment and epigenetic patterns, on child development and health in the context of a developing country metropolitan city (Rio de Janeiro, Southeastern Brazil). To the best of our knowledge, no other similar projects are being performed in the country.

A pilot study was carried out between September 2017 and August 2018 to evaluate the reliability and suitability of the tools, instruments, techniques and methodologies that will be employed in the PIPA Project. This paper presents the learned lessons and strategies to address the shortcomings detected in the pilot study.

### The PIPA project

The PIPA Project research team postulates three hypotheses: a) “Exposure to environmental chemical pollutants determines fetus developmental alterations, causes adverse birth health effects and harms child neuromotor and cognitive development”; b) “The effects of environmental pollutants on fetus and child health are modulated by interactions with sociocultural environment and epigenetic patterns”; c). “Exposure to violence, as a chronic stressor, alters susceptibility to environmental chemical pollutant effects in child health.”

### Design and Study population

The PIPA project is a birth cohort study in which the study population will comprise all pregnant women and their children, born in the Maternity School of the Federal University of Rio de Janeiro (UFRJ), at any gestational age, by either vaginal or cesarean delivery, in a period of 12 months. Eligibility criteria include pregnant women, 16 years old or older, living in the city of Rio Janeiro, where the Maternity School is the reference hospital for delivery. No participant exclusion due to clinical intercurrences during pregnancy or delivery will be performed. All eligible pregnant women will be given the opportunity to participate in the study and will be asked to sign an Informed Consent Term.

The UFRJ Maternity School is one of the reference hospitals monitoring high-risk pregnancies in Rio and is also a referral hospital for low-risk pregnancies from individuals attended at the Rio de Janeiro public health system. The prenatal follow-up of high-risk pregnant women is conducted at this hospital, while low-risk pregnancies are followed up at Family Health Centers close to the pregnant women’s homes.

The hospital is located in the South Zone region of the city and is the delivery reference for eight Family Health Centers (FHCs) responsible for the prenatal assistance care of populations living in low-income communities in this region. The hospital performs 2,000 to 2,500 deliveries/year, about 50% from high-risk pregnancies.

All pregnant women who attended a Family Health Center in the South Zone region of the city for prenatal assistance and the high-risk pregnant women monitored at the University Maternity School, are referred, in their third trimester, to participate in an orientation seminar at the University Maternity School. These seminars consist of lectures on delivery, breastfeeding, and pregnant women’s rights, in addition to a visit to the University Maternity School facilities.

### Data and biospecimen collection

The study protocol includes interviews, physical examinations, and collection of biological samples at the seventh month of pregnancy, at birth, and during the postnatal period until the age of four years old (Table I).

**Table I:** Schedule for Data Collection.

	Prenatal	Birth	3m	6m	12m	24m	36m	48m

**Clinical Exam**								
Anthropometric		×	×	×	×	×	×	×
Physical Exam		×	×	×	×	×	×	×
NCPDTests^1^			×	×	×	×	×	×
**Biological Samples**								
Umbilical Cord Blood		×						
Meconium		×						
Tissue samples^2^		×						
Maternal milk			×	×				
Urine (child)			×	×	×	×	×	×
Urine (mother)	×							
Blood (mother)	×							
Saliva (child)		×	×	×	×	×	×	×
**Questionnaires**								
Gestation: 32^nd^ week	×							
Birth		×						
Follow up		×	×	×	×	×	×	×

^1^ Neuromotor, cognitive and psychologic developmental tests; ^2^umbilical cord and placenta.

The interviews will be performed by the application of a questionnaire (Enrollment Questionnaire; Birth Form; Follow-up Questionnaire) by trained interviewers. The Enrollment Questionnaire was elaborated based on the Norwegian Mother and Child Cohort Study and the Pelotas Birth Cohort Study questionnaires [20,21]. The questionnaire comprises nine groups of questions: Group 1 – ID and contacts; Group 2 – Social, demographic, and economic; Group 3 – Previous morbidities and pregnancy history; Group 4 – Prenatal examination records; Group 5 – Medicine use and drug use (including alcohol and tobacco); Group 6 – Physical activities; Group 7 – Emotional state and Violence approach; Group 8 – Dietary routine; Group 9 – indoor and outdoor environmental and occupational exposure sources to the assessed environmental pollutants. All fathers, if possible, will be also interviewed on specific issues, such as drug use, occupation and medical history.

The study comprises four major components: gestational endpoints, birth endpoints, childhood developmental endpoints and metal, pesticide and plasticizer exposure assessments. Sociocultural conditions and violence exposure are being considered as modifying factors. They will be investigated in each one of the endpoints regarding the adjustments required in the statistical model.

Violence exposure will be determined as urban violence, through local indicators such as homicide mortality rates, gun violence and crime incidents and domestic violence statistics.

Domestic violence will also be evaluated through mother reports. This information will be obtained during the gestational period and throughout the post-natal period. The Registration Questionnaire and the Follow-up Questionnaire will comprise questions approaching physical and verbal aggressions between partners and with the children. Immune-inflammatory biomarkers will be measured in biological samples at birth and during the follow-up (umbilical cord blood and urine).

The PIPA project will investigate the occurrence of DNA methylation patter. This biomarker will be evaluated in the following biological samples: Whole blood samples from mother and umbilical cord; buccal epithelial cells from children saliva samples collected at birth, and annually up to four years old. As the pattern of epigenetic changes is not known, we cannot predict the statistical treatment that will be used. This will be an exploratory analysis of the results.

### Gestational endpoints

Between the 28th and 32nd week of gestation, all pregnant women that agreed to participate in the study will be interviewed and biological samples (blood and urine) will be collected.

The assessed gestational endpoints include blood pressure, glucose and lipid metabolism, endocrine regulation alterations regarding the biological metals and organochlorine compounds (pesticides and polychlorinated biphenyls) concentrations.

### Birth endpoints

A Birth Form will be completed the day after birth by copying birth health records from hospital charts. The following endpoints will be extracted: weight, length, and head circumference at birth; signs of birth asphyxia; placental weight and dimensions; fetal growth information (from ultrasound exam); gestation duration at delivery; congenital malformation information; and neonatal death, or miscarriage or fetal death.

The research team will monitor the pregnant women from the moment they sign the Informed Consent Term until delivery. In the case of fetal loss, miscarriage or neonatal death, an interview will be conducted with the parents to identify the conditions and factors that could have contributed to this outcome.

A physical examination will be performed to repeat the head circumference, weight and length measurements, 24 hours after delivery. Biological samples will be collected at birth (umbilical cord blood) to estimate pollutant concentrations and immune-inflammatory biomarkers.

### Childhood developmental endpoints

**Figure 1:**
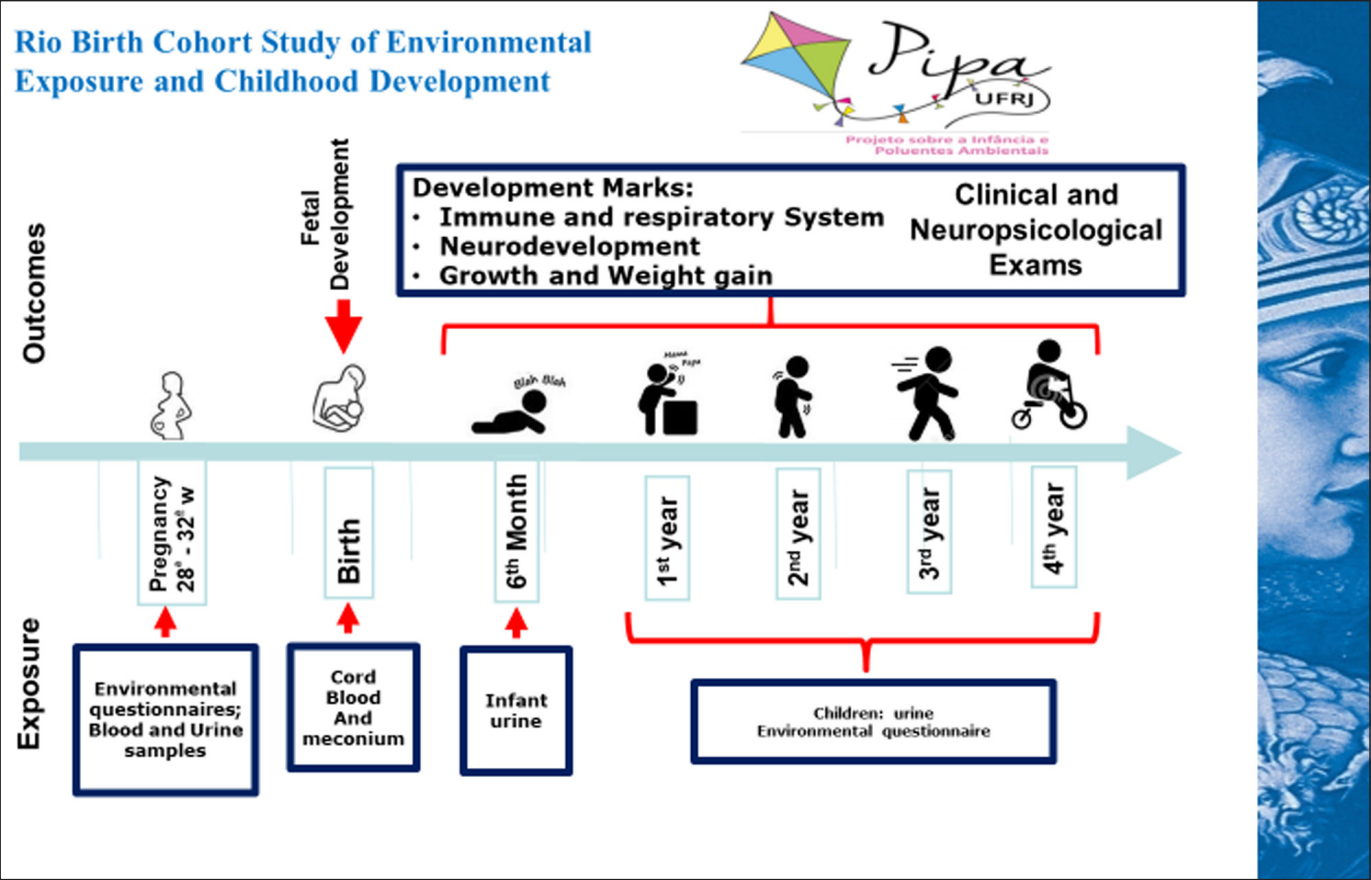
Follow-up endpoints.

Eligible children will be periodically attended at the Maternity School during follow-up visits for clinical monitoring, according to the schedule displayed in Figure 1. During these visits, they will be evaluated by the study team, comprising pediatricians, nurses, social assistants, physiotherapists, psychologists and nutritionists. This evaluation will consist of a follow-up questionnaire application, physical examination and application of neuromotor, cognitive and psychological developmental tests.

The follow-up questionnaire aims to investigate post-natal exposure to environmental pollutants and record child development and health intercurrences. Breastfeeding habits will also be assessed in this questionnaire, focusing on distinguishing exclusive breastfeeding. Breast milk collection will be performed in the 3^rd^ and 6^th^ follow-up months.

Physical examinations will be carried out by trained professionals to evaluate respiratory and neurological systems. The Denver, Alberta and Gesell scales will be applied to evaluate neuromotor, cognitive and psychological development. Childhood developmental endpoints include physical growth alterations (weight, height, waist, abdomen and mid-upper arm circumferences, Body Mass Index), neurodevelopment (short-term memory, speech and language, motor ability, attention, spelling, reading, executive function, non-word repetition) and immunological and respiratory (asthma; allergic sensitization; wheezing with whistling on the chest; coughing during the night) outcomes.

### Exposure assessment to metals, pesticides and plasticizers

The assessed compound concentrations in the collected biological samples will be determined throughout the study, as described in Table II. During the pregnancy, blood and urine from the mother will be obtained, while, maternal milk shall be sampled after delivery in the third and sixth follow-up months. Cord blood will be obtained during the delivery. Child urine will be collected up to four years of age.

**Table II:** Environmental pollutants, types of sample and biological matrices.

Biologic Matrix	Environmental Pollutants	Types of sample

**Prenatal: 27th to 32nd week.**		
Blood	Metals^(1)^; Organochlorine pesticides; Bisphenol; Polychlorinated biphenyls (PCBs); Phthalates;	Parents
Urine	Metals^(1)^ Pyrethroids; Bisphenol; Phthalates.	Parents
**Birth**		
Umbilical Cord Blood	Metals^(1)^ Organochlorine pesticides; Polychlorinated biphenyls (PCBs);	Newborn
Meconium	Metals^(1)^	Newborn
**Postnatal until 4 years old**		
Urine	Metals^(1)^ Pyrethroids; Bisphenol; Phthalates.	Newborn/infant/toddler
Maternal Milk ^(2)^	Metals^(1)^ Organochlorine pesticides; Polychlorinated biphenyls (PCBs).	Mother

^(1)^ Metals: Lead, Arsenic. Cadmium and Mercury. ^(2)^ In the 3^rd^ and 6^th^ follow-up months.

Mother blood samples will be collected using polypropylene vacuum tubes containing EDTA. Cord blood will be collected using disposable 20 mL syringes in polypropylene vacuum tubes containing EDTA, applying a quick transfer time to prevent hemolysis. The collected urine samples will be placed in wide mouth polypropylene flasks. Breast milk samples will be collected and stored in light protected glass flasks in a freezer. All blood, urine and breast milk samples will be aliquoted into small glass tubes and stored at –20 or –80°C as required.

The laboratories of the Institutions comprising this project are accredited by International Accreditation Standards. The laboratory analyses of all samples will be performed meeting all quality assurance measures. The accuracy and precision of all analyses will be evaluated on a regular basis, through external quality assessment programmes and inter laboratorial verifications. One sample will be sent for certified control at every 20–30 samples batch.

Metal concentrations (lead, mercury, arsenic, and cadmium) will be assayed by inductively coupled plasma mass spectrometry (ICP-MS) in maternal and cord blood, in maternal and infant urine, and in maternal milk at the Metal Laboratory belonging to the Evandro Chagas Institute (IEC/Health Ministry, Brazil).

Pyrethroid metabolites will be analyzed in both mother and child urine samples. Organochlorine pesticides and polychlorinated biphenyls will be analyzed in maternal milk, mother and cord blood. These analyses will be performed at the Fiocruz Toxicology Laboratory.

Pyrethroid pesticides will be analysed by indirect assessments of their metabolites, namely 3-PBA (3-phenoxybenzoic acid) and 4-FPBA (4-fluor 3-phenoxy benzoic acid) by solid phase extraction and liquid chromatographic analysis coupled to a sequential mass spectrometer with a triple quadrupole detector [22]. HCH isomers, hexachlorobenzene (HCB), Aldrin, Dieldrin, Endrin, p, p’-DDT, p, p’-DDD, p, p’-DDE, Mirex, Methoxychlor, Endosulfan, Chordane and Heptachlor, as well as 10 PCBs congeners, will also be analysed, adapted from Sarcinelli et al. (2003) [23]

The study also aims to analyze plasticizer concentrations (bisphenol, phthalates) in the collected biological samples and metal concentrations in meconium. However, no laboratory in Brazil has developed the methodologies and technologies necessary for these analyses. Thus, international partnerships will be required to accomplish these determinations. We will also collect and store extra blood and urine samples from our study participants, which may be used in specific studies (nested studies).

### Statistical Analyses

A hierarchical model will be developed to identify and differentiate possible confounding and modifying factors. The confounding factors will be investigated for control during the statistical analyses. The modifying factors will be detected by specific statistical tests and the exposure variables will be adjusted accordingly. The potential modifying variables to be assessed include pre-gestational and gestational morbidities, mainly because of our study population is composed of two groups of pregnant women, those at risk and those presenting low risk. In addition, other factors will be considered as use of alcohol, tobacco and other drugs; use of medications and dietary supplements; sociodemographic characteristics (age, schooling, familiar income, household conditions, domiciliary density); and exposure to violence.

Concerning measured data validation, especially concerning developmental assessments, we will carry out interview repetitions and assessments of child physical and cognitive developments in a randomized subsample of mothers and children of the study population. Kappa and intraclass correlation coefficients will be calculated according to each variable analysis.

The PIPA project encompasses three advisory committees to support project managing and monitoring, namely the Data Security committee, the Ethical committee, and the Publishing committee. It was approved at the UFRJ Maternity School Ethics Committee (reference number: 2.092.440) and at the Fiocruz Foundation Ethics Committee (reference number: 2.121.397).

### The PIPA Pilot Study

The PIPA Pilot Study was performed for 12 months, from September 2017 to August 2018, at the Federal University of Rio de Janeiro Maternity School, with financial support from Brazil Ministry of Health. This pilot study aimed to test and evaluate the research tools, methodologies, techniques and enrollment strategies to be applied in the birth cohort. It also verified the sociodemographic characteristics of the PIPA study population regarding the Rio de Janeiro municipality population.

In October and November 2017, all pregnant women older than 16 who attended an Orientation Seminar at the Maternity School were invited to participate in the Pipa Pilot Study. All those that accepted the invitation signed an Ethical Consent Form. The study population was constituted by these pregnant women and their offspring born during the following months. The newborns were evaluated and monitored since birth until 6 months of age, through clinical and laboratory exams (Table III).

**Table III:** Recruitment and participation in the PIPA Pilot Study.


Enrollment: October and November/2017 (2 months): All pregnant women that attended the Prenatal Maternity School Program/UFRJ: 209 were invited to participate: 142 (67.9%): agreed. Phase 1_PreNatal: 142 pregnant women, 139 (98%) collected blood, 142 (100%) urine and hair samples. Phase 2_Birth: 135 (95, 1%) newborns: 126 (93%): collected umbilical cord blood samples; 54 (40%) collected urine samples; 117 (86.7%) collected meconium samples. Phase 3_Follow-up: 130 eligible newborns: 77 (59%) returns (1^st^, 3^rd^ or 6^th^ months). A total of 177 newborns urine samples and 151 maternal milk samples were collected


During this period, 209 pregnant women attended the Orientation Seminars and 142 (67.9%) accepted to participate in PIPA Pilot study. All participants answered the Enrollment questionnaire and gave urine and hair samples (100%), while 139 (98%) gave blood samples. A total of 135 (95%) children were born, with four twin births, from October 2017 until February 2018, and 11 (7.7%) births were lost because they occurred in another maternity. The Birth Form was filled for all these newborns. Umbilical cord blood samples were obtained from 126 newborns (93%, loss of 7%), meconium samples were obtained from 117 newborns (86.7%, loss of 13.3%), and urine samples were obtained from 54 newborns (40%, loss of 60%). A total of 130 newborns were eligible for follow-up visits (five dropouts occurred). A total of 56 (43%) returns for follow-up visits in the first month were obtained, 67 (51,5%) in the third month and 58 (44,6%) in the sixth month. A total of 53 (41%) children did not attend any clinical evaluation. All other newborns that attended the follow-up visits (77_59%), even if only once (in the 1^st^, 3^rd^ or 6^th^ months), the programmed biospecimens were collected during the follow-up visits: maternal milk (151 samples) and newborn urine (177 samples).

The PIPA pilot study population displays demographic characteristics similar to those from the overall population of the city of Rio de Janeiro (Table IV). However, the PIPA maternal study population is a little older (over 40 years old – 7.2% vs. 3.9%), present more years of study (92.7% vs. 82.5%) and most self-referred as non-white (71.1% vs. 63.4%). This is probably due to the fact that the UFRJ School Maternity is a reference for high-risk pregnancies and that age lower than 16 is an exclusion criterion. A higher number of PIPA Pilot study newborns weighing above 4,000gr (8.3% vs. 5.1%), a lower number of newborns large for gestational age (9.2% vs 15.9%) and a higher boy/girl ratio (57.3/43.7% vs 51.1/48.9%) are noted when compared to newborns from the overall Rio de Janeiro population.

**Table IV:** Comparison between the PIPA Pilot Study population data and Rio de Janeiro municipality population data.

Maternal Characteristics		PIPA Pilot Study		MRJ*	

		%	(N)	%	(N)

**Maternal age (139)^(1)^**	**15 to 19 years old**	8.6	(12)	13.9	(11194)
	**20 to 39 years old**	84.2	(117)	82.2	(66024)
	**Up to 40 years old**	7.2	(10)	3.9	(3154)
**Years of Study (137)^(1)^**	**Lower than 8 years of study**	7.3	(10)	1.5	(13724)
	**Higher than 8 years of study**	92.7	(127)	82.5	(64494)
**Ethnicity (135)^(1)^**	**White**	23.9	(34)	36.6	(29057)
	**Non-White**	71.1	(101)	63.4	(50230)
**Newborn Characteristics**		**PIPA Pilot Study**		MRJ*		

		**%**	**(N)**	**%**	**(N)**

**Gender**	**Male**	57.3	(75)	51.1	(42,223)
	**Female**	43.7	(56)	48.9	(40,370)
BirthWeight ^(1)^**	**1.000–2500**	9	(12)	8.6	(7,143)
	**2500–3999**	82.7	(110)	86.2	(71,224)
	**> 4000**	8.3	(11)	5.1	(4,234)
Birth Weight Adequacy for Gestational Age^(1)^***	**SGA**	9.2	(11)	9.3	(165)****
	**AGA**	81.5	(97)	74.8	(1,324)****
	**LGA**	9.2	(11)	15.9	(282)****

* MRJ: Rio de Janeiro Municipality. Source: MS Datasus/Sinasc–2016. ** Total PIPA number: 133 newborns; Total number RJ: 82.601 newborns. *** Total PIPA number: 119 newborns. **** Source: Kale et all, 2018 [25]; Total live births: 1,771; SGA: Small for the gestational age; AGA: adequate for the gestational age; LGA: large for the gestational age. ^(1)^ Incomplete data on the specific covariates.

The detection rate of analyzed metals (arsenic, mercury, cadmium, and lead) in both mother and cord blood samples was of 100% (Table V). For pyrethroid pesticide metabolites (Table VI), the detection rate was 47.9% (3PBA metabolite) in mother urine samples, while newborn urine samples presented a detection rate of over 20% at birth and during the follow-up. The metabolite 4FPBA, more specific for cyfluthrin, presented a detection rate of 10% among mothers, and no detection in babies.

**Table V:** Metal levels in mother and umbilical cord blood – Pilot study.

Metals	Sample	Geometric Mean (95% IC)	Min	Max

**Pb (µg/dL)**	Mother blood	3.74 (3.40–4.12)	1.11	15.26
	Umbilical Cord Blood	3.85 (3.53–4.19)	1.43	16.03
**Hg (µg/L)**	Mother blood	1.00 (0.85–1.18)	0.33	13.32
	Umbilical Cord Blood	1.11 (0.97–1.27)	0.35	6.38
**Cd (µg/L)**	Mother blood	0.30 (0.29–0.53)	0.002	22.43
	Umbilical Cord Blood	0.41 (0.32–0.52)	0.004	17.41
**As (µg/L)**	Mother blood	10.27 (9.37–11.18)	0.33	36.48
	Umbilical Cord Blood	10.31 (9.75–10.93)	4.89	19.94

Metal limits of detection: Pb 0.015 μg/L; Hg 0.007 μg/L; Cd 0.002 μg/L; As 0.003 μg/L.

**Table VI:** 3BPA and 4FBA metabolite detection rates – Mother and child urine.

Samples	N analysed	3PBA ng mL-1 Detection		4FPBA Detection	

		N	Rate	N	Rate

Mother Urine	140	67	47.9%	14	10%
Urine newborn	34	8	23.5%	0	0
Urine 1^st^ follow up	26	5	19.2%	0	0
Urine 2^nd^ follow up	50	14	28%	0	0
Urine 3^rd^ follow up	56	12	21.4%	0	0

Metabolite limits of detection: 3BPA 0.06 ng mL–1; 4FPBA 0.05 ng mL–1.

## PPIA Project: The Lessons Learned from the Pilot Study

### Strength and limitations

The PIPA project is the first birth cohort in Brazil, focusing on the investigation of environmental pollutant exposure effects on maternal-child health since gestation. It is a hospital-based cohort study developed at a university hospital (UFRJ Maternity School) with estimates of 2,000 to 2,500 deliveries/year. The PIPA project study population is relatively small to determine the multiple possible effects of environmental pollutant exposure on maternal/fetal/child health. Nevertheless, a wide range of biomaterials will be collected, such maternal and umbilical blood, maternal and newborn urine and meconium, as well as maternal milk, which will allow for multiple possibilities of investigation and may provide a foundation for future studies concerning the toxic effects of environmental pollutants on maternal/fetal/child health.

During the PIPA Pilot study, many lessons were learned by the research team. We consider that the main problem was participant returns to the follow-up visits. We observed the requirement of altering our data collection scheme, excluding the first-month follow-up visit, based on information obtained from the mothers about the logistic difficulties concerning family displacement in this moment. We also observed the need to help the participants with displacement costs, improve our digital tools and use social media to build a reliable liaison between the research team and project participants.

As an engagement strategy for mothers and their babies to enroll in this study, the project team has developed a “Health, Environment and Child Development Educational Program” at the Family Health Centers and at the Maternity School. It will focus on educational activities related to maternity, fetus development and child health using digital interactive tools, including a web site and a mobile app.

During the data analysis, we observed data incompleteness of some covariables. This is probably due to the fact that the pilot study data collection was performed through printed questionnaires applied by trained interviewers, followed by database insertion. This process was monitored and checked by a supervisor but, unfortunately, some data were lost in the process. To deal with this and improve data completeness, a specific database system is being developed for the PIPA project. The questionnaires and biospecimens will be identified with a unique ID and barcode, respectively, which will trace the laboratory analyses. The questionnaire data will be inserted into the database system, using a single data entry with response verification performed periodically.

All professionals involved in the data collection are being trained and periodically evaluated concerning questionnaire application and clinical and anthropometric evaluations. Measurement quality will be estimated by interview and anthropometric evaluation repetitions in a mother-child subsample. Kappa and intraclass correlation coefficients will be calculated according to each variable analysis.

In the pilot study, the analysis of the gestational diabetes melittus (GDM) variable was performed by global agreement, with the free-marginal multirater kappa calculated [24]. The information collected from the mother regarding the presence of GDM was compared to the information collected from the medical record. This comparison was made for 127 pregnant women, (medical records were not available for 15 pregnant women). A global agreement of 91.34% was observed, with a Kappa index of 0.83 (95% CI 0.73–0.92), considered excellent.

The PIPA project is integrated within the healthcare system of the study region. This condition allows for the sensibilization of local government decision makers concerning the adoption of instruments and techniques for the investigation of environmental pollutant exposure during prenatal assistance care, in addition to health personnel capacitation, in order to expand the research to other maternities attending other regions of the study municipality.

The significant challenges of the PIPA study include funding, used to maintain the research and the cohort, as well as the families’ full participation, while limiting biases that might impact the internal validity of the study. Table VII displays the risk points (challenges) for developing the PIPA study and the strategies that the research team has been developing to deal with them.

**Table VII:** PIPA Project Risk Points and Responding Strategies.

Risk points	Responding strategies

**Management structure**	
Underestimating of research costs	Permanent search for financial support Careful evaluation of the Pilot Study
Underestimating number of required staff High research assistant and interviewer turnover	Permanent hiring and training
Laboratory capacity underestimation Absence of laboratorial capacity regarding some specific technical analyses	Searches for other laboratories with similar qualifications Searches for international partnerships
Establishment of an infrastructure to attend and monitor the population during the cohort period	Accomplishment of alternative attendance schemes for using non-occupied periods
High local government decision makers turnover	Development and maintenance of ongoing collaboration and information nets between the researchers, the maternity and family health center teams
**Participant recruitment**	
Living far from Maternity: monetary displacement costs	Monetary provision to help the participants with displacement costs to the Maternity School, during the follow-up period
No time to answer the questionnaire and to collect the biological samples	Review of the Enrollment questionnaire and Seminar dynamics to optimize data collection. Maximize participant convenience: data collection efficiency; data collector flexibility.
**Participant retention**	“Health, Environment and Child Development Educational Program”
Faults in the follow-up visits	Ongoing sensibilization and contact Establishment of a reliable liaison between the research team and project participants Use of digital tools: social nets, PIPA website, interactive communication Maintain communication: phone calls, messages. Obtain other contact detail information;
**Data Collection**	Daily tracking of participants approaching due dates Reminders through direct communication in each data collection time-point Rescheduling appointments Ongoing interviewer training Recruiting specialized teams to collect biological samples
**Quality Assurance**	
**Incompleteness of covariate data**	Rigorous information system infrastructure Questionnaire information collected directly within the database system, using a single data entry with periodic response verification Periodic data tracking and quality checks
	Unanimous adaptation of pre-specified data collection and management protocols Collecting data relating to numerous potential confounding variables Careful control through statistical analyses

### Perspectives

One of the unique features of the PIPA project is the main goal of investigating environmental pollutant exposure during pregnancy and the effects on maternal/child/fetus health. It is the only birth cohort in Brazil up to now based on this core issue. The results of the study may not be applicable to the overall Rio de Janeiro population, or to the Brazilian population, as the study is not population-based. Nevertheless, it proposes a comprehensive assessment of pre- and postnatal exposure to environmental chemicals at multiple time points, in a population living in a highly urbanized developing country. It will also provide important information on several hypotheses related to prenatal exposure to environmental chemicals and potential adverse health effects for pregnant women and their children.

The accomplishment of the pilot study from 2017 to 2018 allowed the research team to learn about the strengths and limitations of the study and take actions necessary to fix or minimize them. The PIPA pilot study population has similar socioeconomic characteristics to the Rio de Janeiro population and the PIPA project is integrated with the healthcare system of the study region, which may, in turn, use the PIPA results in the elaboration of public health politics addressed for the prevention of the environmental pollutant exposure during pregnancy, as well for population information.

Finally, one of the more important advantages of the PIPA project is the financial support from the Brazilian Government and its accomplishment by a research team originated from two respected learning and research public Brazilian institutions. The PIPA project intends to be a guideline for the development of country-specific pollution exposure protective actions during gestation and childhood, as well as a basis for the implementation of public health politics aiming to promote maternal and infant health.
